# MicroRNA composition of plasma extracellular vesicles: a harbinger of late cardiotoxicity of doxorubicin

**DOI:** 10.1186/s10020-022-00588-0

**Published:** 2022-12-14

**Authors:** Justyna Totoń-Żurańska, Joanna Sulicka-Grodzicka, Michał T. Seweryn, Ewelina Pitera, Przemysław Kapusta, Paweł Konieczny, Leszek Drabik, Maria Kołton-Wróż, Bernadeta Chyrchel, Ewelina Nowak, Andrzej Surdacki, Tomasz Grodzicki, Paweł P. Wołkow

**Affiliations:** 1 Center for Medical Genomics OMICRON, Jagiellonian University Medical College, ul. Kopernika 7C, 31-034 Krakow, Poland; 2 Department of Rheumatology, Jagiellonian University Medical College, Krakow, Poland; 3 Medical College and John Paul II Hospital, Jagiellonian University, Krakow, Poland; 4 Department of Pharmacology, Jagiellonian University Medical College, Krakow, Poland; 5 Second Department of Cardiology, Jagiellonian University Medical College, Krakow, Poland; 6 Department of Internal Medicine and Gerontology, Jagiellonian University Medical College, Krakow, Poland; 7 Department of Cancer Biology and Genetics, Center for Pharmacogenomics, College of Medicine, The Ohio State University, Columbus, OH USA

**Keywords:** Clinical transcriptomics, Micro-RNAs (miRNAs), Extracellular vesicles (EVs), Cardiotoxicity, Doxorubicin, Childhood acute lymphoblastic leukemia (ALL)

## Abstract

**Background:**

The use of doxorubicin is associated with an increased risk of acute and long-term cardiomyopathy. Despite the constantly growing number of cancer survivors, little is known about the transcriptional mechanisms which progress in the time leading to a severe cardiac outcome. It is also unclear whether long-term transcriptomic alterations related to doxorubicin use are similar to transcriptomic patterns present in patients suffering from other cardiomyopathies.

**Methods:**

We have sequenced miRNA from total plasma and extracellular vesicles (EVs) from 66 acute lymphoblastic leukemia (ALL) survivors and 61 healthy controls (254 samples in total). We then analyzed processes regulated by differentially expressed circulating miRNAs and cross-validated results with the data of patients with clinically manifested cardiomyopathies.

**Results:**

We found that especially miRNAs contained within EVs may be informative in terms of cardiomyopathy development and may regulate pathways related to neurotrophin signaling, transforming growth factor beta (TGFβ) or epidermal growth factor receptors (ErbB). We identified vesicular miR-144-3p and miR-423-3p as the most variable between groups and significantly correlated with echocardiographic parameters and, respectively, for plasma: let-7g-5p and miR-16-2-3p. Moreover, vesicular miR-144-3p correlates with the highest number of echocardiographic parameters and is differentially expressed in the circulation of patients with dilated cardiomyopathy. We also found that distribution of particular miRNAs between of plasma and EVs (proportion between compartments) e.g., miR-184 in ALL, is altered, suggesting changes within secretory and miRNA sorting mechanisms.

**Conclusions:**

Our results show that transcriptomic changes resulting from doxorubicin induced myocardial injury are reflected in circulating miRNA levels and precede development of the late onset cardiomyopathy phenotype. Among miRNAs related to cardiac function, we found vesicular miR-144-3p and miR-423-3p, as well as let-7g-5p and miR-16-2-3p contained in the total plasma. Selection of source for such studies (plasma or EVs) is of critical importance, as distribution of some miRNA between plasma and EVs is altered in ALL survivors, in comparison to healthy people, which suggests that doxorubicin-induced changes include miRNA sorting and export to extracellular space.

**Supplementary Information:**

The online version contains supplementary material available at 10.1186/s10020-022-00588-0.

## Introduction

Anthracyclines, including doxorubicin, have contributed to improved survival in childhood acute lymphoblastic leukemia (ALL) from less than 10% to 90% and are still the most widely used antineoplastic drugs worldwide (Birch et al. 1988; Bonaventure et al. 2017). However, because of the lack of specificity for cancer cells, anthracyclines can also damage healthy, non-cancer cells, causing severe complications, including cardiotoxicity, during chemotherapy, as well as many years after treatment cessation (Amigoni et al. 2010; Biancaniello et al. 1980). Among multiple health problems, heart disease is the most common non-cancer related cause of death among cancer survivors. Cardiac mortality in childhood cancer survivors is 3.4 times higher than in general population, and the number of excessive cardiac deaths increases with time (Fidler et al. 2017; Mertens et al. 2008). Lipschultz et al. indicated that more than 50% of all doxorubicin-treated ALL survivors exhibit abnormalities of left ventricular afterload or heart muscle contractility (Lipshultz et al. 1991) several years after treatment cessation. Other research has revealed that myocardial atrophy, ventricular mass reductions and heart dilation are the main contributors to heart problems developing due to anthracycline use in cancer patients (Jordan et al. 2018; Kajihara et al. 1986). Moreover, patients suffering from cardiomyopathy caused by doxorubicin have worse prognosis than those with idiopathic or ischemic heart-caused forms of the disease.

Von Hoff et al. identified total cumulative dose of the drug as the main contributor to the doxorubicin-caused heart failure (Hoff et al. 1979). The central dogma of anthracyclines evoked cardiomyopathy, based on acute doxorubicin action, points to oxidative stress caused by excessive amounts of reactive oxygen species (ROS) produced due to severe functional disruption of mitochondria (Octavia et al. 2012; Berthiaume and Wallace 2007; Thayer 1988). Doxorubicin has also been shown to induce disturbance in calcium signaling and changes in expression of cardiomyocyte genes (Solem et al. 1996; Ito et al. 1990). In addition, doxorubicin administration brings about massive DNA damage, including 8-oxoguanine formation, DNA intercalation, and topoisomerase 2 poisoning, with downstream double-strand breaks (DSBs) formation (L’Ecuyer et al. 2006; Qiao et al. 2020; Marinello et al. 2018). As DNA lesions are repaired only partially (Qiao et al. 2020), these should have immediate consequences at the transcriptomic level and may be considered as the cause of long-term treatment side effects manifested at distant time points. However, knowledge on long-term transcriptomic processes leading to health complications due to anthracycline use is very limited, despite constantly increasing number of cancer survivors (Miller et al. 2019). Furthermore, the analysis of gene expression in cardiac tissues is not feasible in human subjects, thus studies on molecular aspects of doxorubicin action are mostly limited to cultured cardiomyocytes in a short time scale or to animals (Wan et al. 2021).

In recent years the question on usability of blood circulating factors in heart disease progression monitoring has been raised (Stadiotti et al. 2019). It has been demonstrated that circulating miRNA may be predictors of sudden cardiac/arrhythmic death in patients with coronary artery disease (Silverman et al. 2020). Akat et al., for example, show that heart- and muscle-specific circulating miRNAs increased up to 140-fold in advanced heart failure, whereas in stable heart disease, fold changes were lower, hence, miRNAs could serve as indicators of heart muscle injury (Akat et al. 2014).

It is known that miRNAs may circulate in blood in a form of protein-bound complexes, but can also be encapsulated in extracellular vesicles (EVs), and each of these compartments may contain a different set of miRNAs or the same miRNAs, albeit with differing quantities (Endzeliņš et al. 2017). The latest reports on the role of EVs in intercellular communication underscore the stability of miRNAs in EVs and its usefulness as indicators of diverse processes (Jeong et al. 2021). Recently, the role of miRNA encapsulated in extracellular vesicles in cardiac remodeling upon stress has also been emphasized (Tian et al. 2020).

The selective nature of sorting of miRNA into EVs and the surface protein mediated specificity of EVs targeting to recipient cells (Hoshino et al. 2015; Sancho-Albero et al. 2019; Sanz-Rubio et al. 2018) prompt us to look for existence of differences in miRNA expression in both plasma and EVs between ALL survivors treated with doxorubicin and healthy controls. Here, we test the hypothesis that doxorubicin-induced tissue injury is responsible for dysregulation of the transcriptional network, which drives long-term side effects of anthracyclines and which manifests in altered circulating miRNA expression.

## Materials and methods

### Study cohort

The survivor population was recruited from the Childhood Cancer Survivorship Clinic at the University Hospital in Kraków. Informed consent was obtained in accordance with the Declaration of Helsinki. The study was approved by the Bioethics Committee at the Jagiellonian University (Approval No. 122.6120.274.2015). Eligibility criteria included the following: (1) diagnosis of ALL before 18 years of age and (2) 5 or more years since the completion of cancer treatment (doxorubicin). Exclusion criteria included the following: (1) time from the end of therapy for ALL shorter than 5 years, (2) relapse or secondary cancer at the time of the study or during the 5 preceding years. The study participants underwent a comprehensive clinical evaluation, including a physical examination accompanied by anthropometric assessments. Healthy controls were recruited at the Blood Donation Center in Kraków, Poland. Blood sampling, biochemical analyses, and echocardiographic evaluation were performed as previously described (Sulicka-Grodzicka et al. 2019).

### Isolation and characterization of EVs

Plasma EVs were isolated with the miRCURY Exosome Isolation Kit (Exiqon, Qiagen, Aarhus, Denmark) according to the manufacturer’s protocol. Size distribution of EVs was measured by Nanoparticle Tracking Analysis with NanoSight (Malvern Panalytical, Malvern, United Kingdom).

### RNA extraction and preparation of miRNA libraries

Small RNA was extracted from EVs and total plasma with miRCURY RNA Isolation Kit (Exiqon, Qiagen). Libraries were prepared with NebNext Small RNA Library Prep (New England Biolabs, Ipswich, MA, USA). Quality control steps for libraries were performed on TapeStation (Agilent Technologies, Santa Clara, CA, USA) before and after size selection. cDNA concentration was measured using the Quantus fluorometer (Promega, Madison, Wisconsin, USA). Pooled libraries were sequenced with High Output v2.0 reagents on the NextSeq 500 sequencer (Illumina, San Diego, CA, USA).

### miRNA-seq analysis

Demultiplexed sequenced reads from pooled libraries were quality checked via FastQC software, v0.11.8 [135]. The reads were then trimmed to remove primers and poor-quality bases with Cutadapt, v1.18 [72]. Reads with length < 18 or > 30 nucleotides and reads without 3′ adapter were removed. The cleaned reads were then aligned to miRBase database v22.1 [57] and counted using miRDeep2 software v0.0.8 [27]. Normalized miRNA read count generated from miRDeep2 was applied in the subsequently undertaken differential expression analysis. The raw sequences, along with raw and normalized counts from miRDeep2 software were deposited in GEO (GSE145176).

### Statistical methods

All statistical analyses and filtering steps were performed in R (v3.5.2). Briefly, only miRNAs with expression in at least one sample were employed in the statistical analysis. The differential expression was analyzed by the edgeR package with two different experimental designs applied. The first model (xp ~ compartment + status:compartment) was used to test differences in miRNAs expression between ALL survivors and controls in plasma and EVs separately. The second model (xp ~ status + compartment:status) was employed to test whether miRNAs are differentially distributed between plasma and EVs in ALL survivors with respect to controls. Only results with FDR < 0.05 were considered significant. The KEGG, GO, and hallmark enrichment analysis was performed with ‘RbiomirGS’ and ‘clusterProfiler’ R packages. Briefly, we initiated ‘RbiomirGS’ to find target genes for differentially expressed miRNAs in each analysis using various predictive algorithms from the multiMIR database v2.1. We then conducted a logistic regression-based gene set enrichment in ‘RbiomirGS’ to find significant KEGG terms. Since ‘RbiomirGS’ considers the change in expression (logFC), for GO and hallmark enrichment analysis in ‘clusterProfiler’ we divided the significant results from each set on up and down-regulated miRNA. To keep GO analysis transparent, we only showed terms between levels 4 and 9, and terms with FDR < 0.05 alone were considered significant. The correlation between DE miRNA and echocardiographic parameters was tested with the ‘Hmisc’ package and then plotted with the ‘pheatmap’ package. To test in an unbiased fashion whether the expression in plasma/exosomes of miRNA correlates with selected echocardiographic parameters, we applied normalized pseudocounts and Levene’s test to select miRNAs that have the highest variance between cases and controls in plasma or EVs. For further analyses, we used only the ones that remained significantly differentially variable with FDR < 0.05. We then performed correlation analysis with echocardiographic parameters similar to DE miRNAs.

## Results

### Characteristics of the studied groups

There was no statistically significant difference in sex and there was a borderline nonsignificant difference in age between the 66 ALL survivors and 61 healthy blood donors. Subsequently, we used the results of complete blood count and lipid panel tests to compare the two study groups (as presented in Table 1). Additionally, for the ALL cohort, we collected echocardiographic data. Detailed information about echocardiographic parameters of ALL survivors was presented in our previous publication (Sulicka-Grodzicka et al. 2019).

**Table 1:** Basic characteristics of the study groups

	Controls (n = 61)	ALL (n = 66)	p-value
Sex, F/M ratio	1.34	1.35	0.981
Age, years	23 (18–42)	22 (18–38)	0.056
Creatinine (µmol/l)	**76 (48–118)**	**68 (42–97)**	**9.0E−3**
Total cholesterol (mmol/l)	4.50 (2.49–6.60)	4.2 (3.1–5.9)	0.146
HDL (mmol/l)	1.71 (0.93–2.66)	1.67 (0.93–2.79)	0.362
LDL (mmol/l)	**2.69 (1.02–4.89)**	**2.2 (1.2–4.1)**	**1.5E−4**
Triglycerides(mmol/l)	**0.94 (0.31–3.49)**	**0.80 (0.34–2.40)**	**1.4E−3**
RBC (10/µl)	4.95 (4.06–5.92)	4.98 (4.02–5.68)	0.877
Hemoglobin (g/dl)	14.1 (12.1–17.1)	14.95 (12.50–16.90)	0.100
Hematocrit (%)	42.5 (37.0–51.1)	43.5 (36.0–47.7)	0.794
MCV (fl)	87.1 (75.5–94.1)	87.4 (79.0–94.3)	0.639
MCH (pg)	**29.2 (24.4–31.8)**	**29.9 (26.2–33.2)**	**2.0E−03**
MCHC (g/dl)	**33.3 (31.3–35.3)**	**34.4 (32.2–36.5)**	**1.8E−08**
Platelets (10^3^/µl)	321 (147–377)	266 (168–415)	0.547
MPV (fl)	**8.0 (4.3–10.1)**	**10.7 (8.8–12.7)**	**< 2.2E−16**
White blood cells (10^3^/µl)	5.88 (4.04–11.30)	5.48 (2.64–13.04)	0.426
Neutrophils (10^3^/µl)	3.40 (1.83–7.00)	3.05 (1.20–12.20)	0.483
Lymphocytes (10^3^/µl)	1.7 (1.1–2.7)	1.6 (0.3–3.2)	0.066
Monocytes (10^3^/µl)	0.5 (0.22–1.00)	0.5 (0.2–1.1)	0.516
Eosinophils (10^3^/µl)	0.1 (0.0–0.7)	0.1 (0.0–0.5)	0.090
Basophils (10^3^/µl)	**0.0 (0.0–0.1)**	**0.0 (0.0–0.1)**	**0.010**

The median (range) values are reported; the p-value corresponds to the Fisher’s exact test or Wilcoxon signed-rank test for equality of location parameters in the two groups.* P*-values marked with bold indicate statistically significant (*p*-value < 0.05) differences between the groups.

*HDL* high-density lipoproteins, *LDL* low-density lipoproteins, *RBC* red blood cells, *MCV* mean corpuscular volume, *MCH* mean corpuscular hemoglobin, *MCHC* mean corpuscular hemoglobin concentration, *MPV* mean platelet volume

### Extracellular vesicles characteristics

Nanoparticle Tracking Analysis (NTA) analysis revealed that the median vesicle size was 69.75 µm (65.05–73.35) and their concentration was 9.17E+12 particles/ml (5.58E+12–1.46E+13). There were no significant differences between control and ALL groups in particle size and concentration, as the Wilcoxon signed-rank test indicated.

### Differential expression of miRNAs in blood plasma and exosomes

**Fig. 1:**
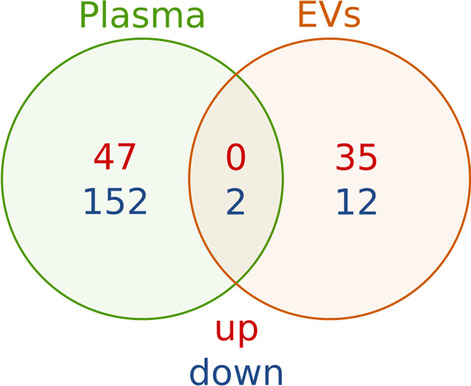
Venn diagram of differentially expressed miRNAs between ALL survivors and control in plasma, EVs. The red color indicates up-regulated miRNAs, whereas the blue color indicates the down-regulated miRNAs

The miRNA sequencing was performed in 61 ALL survivors and 59 control subjects. Due to insufficient RNA amount, 7 samples were excluded from analysis. After removal of unexpressed miRNAs, 1986 miRNAs-precursor pairs were identified in plasma samples and EVs altogether. To better understand the role of miRNAs in ALL survivors, we decided to compare miRNA expression in plasma and EVs separately. The comparison of ALL cases and controls allowed the detection of 201 miRNAs in plasma (Additional file 1) and 49 miRNAs in EVs (Additional file 2). The top 10 miRNAs differentially expressed in plasma and EVs between control and ALL survivors are shown in Table 2 (in the top and middle panels, respectively). Only 2 miRNAs with reduced expression (miR-500a and miR-500b) were common to blood plasma and EVs (Fig. 1).

**Table 2:** The top 10 differentially expressed miRNAs between controls and ALL survivors in blood plasma (top panel), EVs (middle panel) and differentially distributed between these compartments (bottom panel)

miRNA	Precursor	logFC	logCPM	p-value	FDR
Plasma					
miR-184	mir-184	− 5.358	8.897	2.49E−16	4.95E−13
miR-324-5p	mir-324	− 3.276	1.897	4.62E−10	4.59E−07
miR-4753-5p	mir-4753	− 3.149	1.352	2.08E−09	1.05E−06
let-7g-5p	let-7g	− 0.701	13.642	2.11E−09	1.05E−06
miR-579-5p	mir-579	− 3.385	1.968	4.17E−09	1.66E−06
miR-1-3p	mir-1-2	− 2.993	7.536	6.95E−09	2.16E−06
miR-1-3p	mir-1-1	− 2.973	7.480	7.61E−09	2.16E−06
miR-3140-3p	mir-3140	− 3.108	1.629	1.88E−08	4.67E−06
miR-3939	mir-3939	− 2.335	1.270	2.82E−07	6.21E−05
miR-1273c	mir-1273c	4.799	3.364	4.13E-07	8.21E−05
EVs					
miR-221-5p	mir-221	3.766	4.657	3.26E−11	6.48E−08
miR-199a-3p	mir-199a-2	1.722	9.299	9.30E−10	4.63E−07
miR-199b-3p	mir-199b	1.722	9.298	9.31E−10	4.63E−07
miR-199a-3p	mir-199a-1	1.722	9.298	9.32E−10	4.63E−07
miR-203a-3p	mir-203a	3.924	5.776	1.45E−09	5.77E−07
miR-574-5p	mir-574	3.101	3.979	1.48E−08	4.89E−06
miR-148a-5p	mir-148a	− 2.639	5.911	2.00E−08	5.68E−06
miR-200a-3p	mir-200a	2.288	8.178	2.78E−08	6.89E−06
miR-145-5p	mir-145	6.219	4.239	3.27E−08	7.21E−06
miR-378i	mir-378i	1.891	6.074	1.07E−07	2.13E−05
Differentially distributed between plasma and EVs					
miR-184	mir-184	− 6.218	8.897	7.60E−14	1.51E−10
miR-1-3p	mir-1-2	− 5.072	7.536	1.19E−12	1.18E−09
miR-1-3p	mir-1-1	− 4.935	7.480	3.52E−12	2.33E−09
miR-548am-5p	mir-548am	7.756	3.824	3.68E−08	1.75E−05
miR-548o-5p	mir-548o-2	7.625	3.824	5.56E−08	1.75E−05
miR-548c-5p	mir-548c	7.625	3.824	5.58E−08	1.75E−05
miR-208b-3p	mir-208b	7.869	1.993	6.18E−08	1.75E−05
miR-199b-3p	mir-199b	− 1.982	9.298	4.26E−07	9.02E−05
miR-199a-3p	mir-199a-1	− 1.982	9.298	4.26E−07	9.02E−05
miR-199a-3p	mir-199a-2	− 1.977	9.299	4.54E−07	9.02E−05

*logFC* log fold change, *logCPM* log counts per million, *FDR* false discovery rate

Additionally, we investigated whether miRNAs are differentially distributed between the plasma and EVs in ALL survivors with respect to controls (Additional file 3). This analysis showed that particular miRNAs in ALL survivors are preferentially enriched in plasma or in EVs compartments, in comparison to healthy controls. We discovered 95 miRNAs, of which 73 were plasma-specific (logFC > 0) and 22 were EVs-specific (logFC < 0) (Additional file 3). Table 2 (bottom panel) lists the top 10 results for differentially expressed miRNAs in blood plasma and EVs, as well as the miRNAs differentially distributed between these compartments.

### KEGG and GO enrichment analysis

**Fig. 2:**
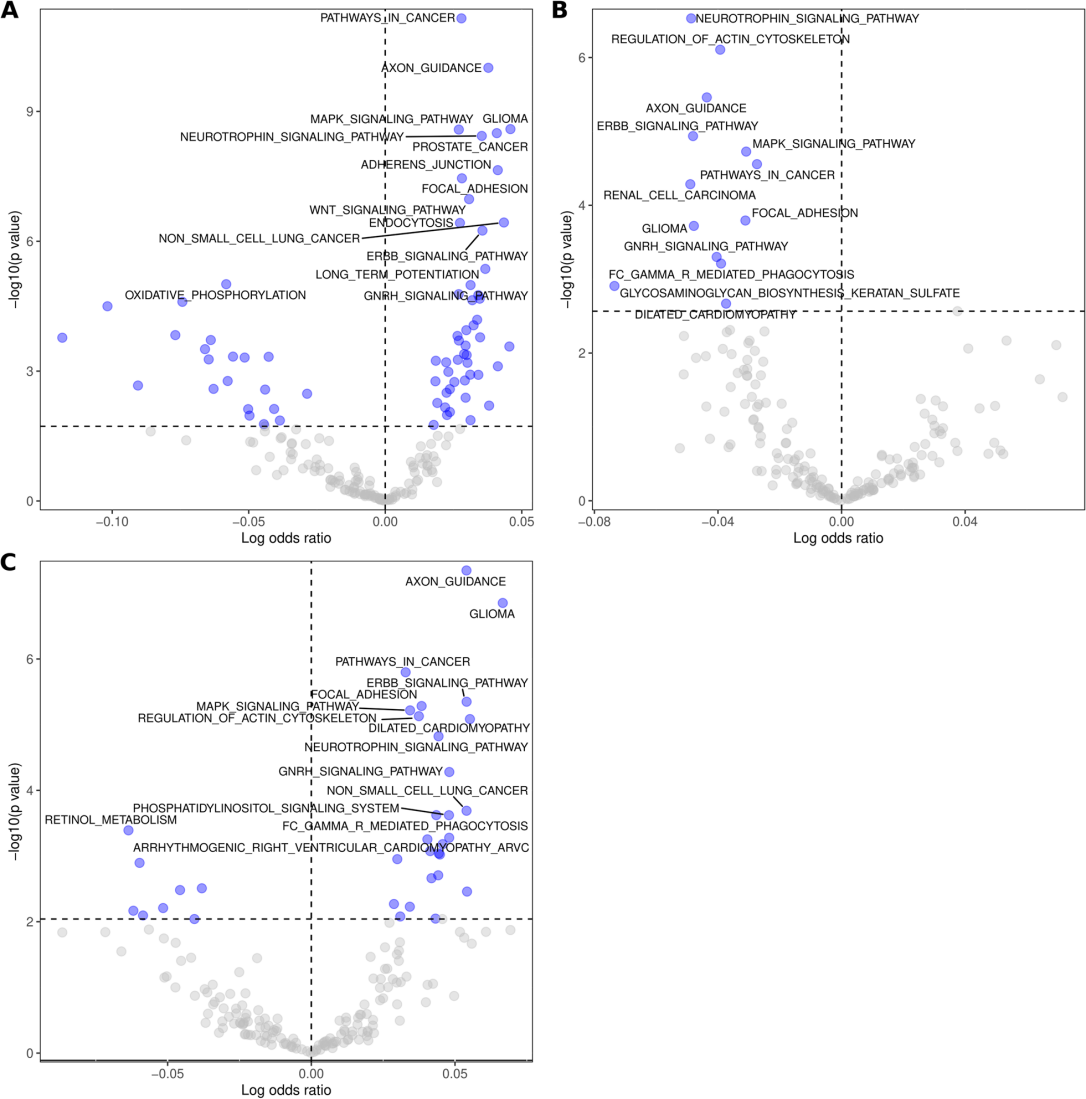
Results of the KEGG enrichment analysis among the targets of differentially expressed miRNAs in plasma (**A**), extracellular vesicles (**B**), and between the compartments (**C**)

To gain insight into the potential functional role of global miRNA expression changes, we performed KEGG pathway analysis for target genes of significant miRNAs in plasma, EVs, as well as differential distribution analysis, taking into account the magnitude of change between tested conditions (logFC). The top 15 enriched KEGG terms of this analysis are shown in Fig. 2. Details of all significant KEGG pathways can be found in Additional file 4. Not surprisingly, a higher number of enriched terms were found for the plasma set, as more differentially expressed miRNAs were identified. Among the 15 most prominent pathways in each case, many were related to or strongly associated with cardiomyopathies, such as ‘axon guidance, ‘MAPK’, ‘ErbB’, ‘regulation of actin cytoskeleton’ and ‘neurotrophin ‘signaling. Interestingly, these have previously been associated with cardiac function or the effects of anthracycline actions. However, the term ‘dilated cardiomyopathy’ appeared only in the KEGG analysis of EVs (Fig. 2B), whereas dilated cardiomyopathy, as well as arrhythmogenic right ventricular cardiomyopathy terms, are present in the analysis, which corresponds to miRNA differentially distributed between plasma and EVs in cancer survivors with respect to controls (Fig. 2C). This supports the hypothesis that anticancer therapy-initiated processes may influence miRNA secretion to the extracellular environment and that miRNA in these two compartments may have a different role and target cells. Moreover, alterations in miRNA secretion to the extracellular environment may have a particularly significant role in the development of cardiac complications.

Next, for the three sets of miRNAs defined above, we performed GO term enrichment analysis, for up- and down-regulated miRNAs separately. For plasma, we detected 212 and 93 enriched terms for up- and down-regulated miRNAs, respectively, while for a set of EVs, we detected 195 and 127 enriched terms, respectively (Additional file 5). As for differentially distributed miRNAs, we detected 123 terms for upregulated miRNAs, which represent plasma-specific distribution, whereas 181 terms for downregulated miRNAs, which represent EVs-specific distribution (Additional file 5).

**Fig. 3:**
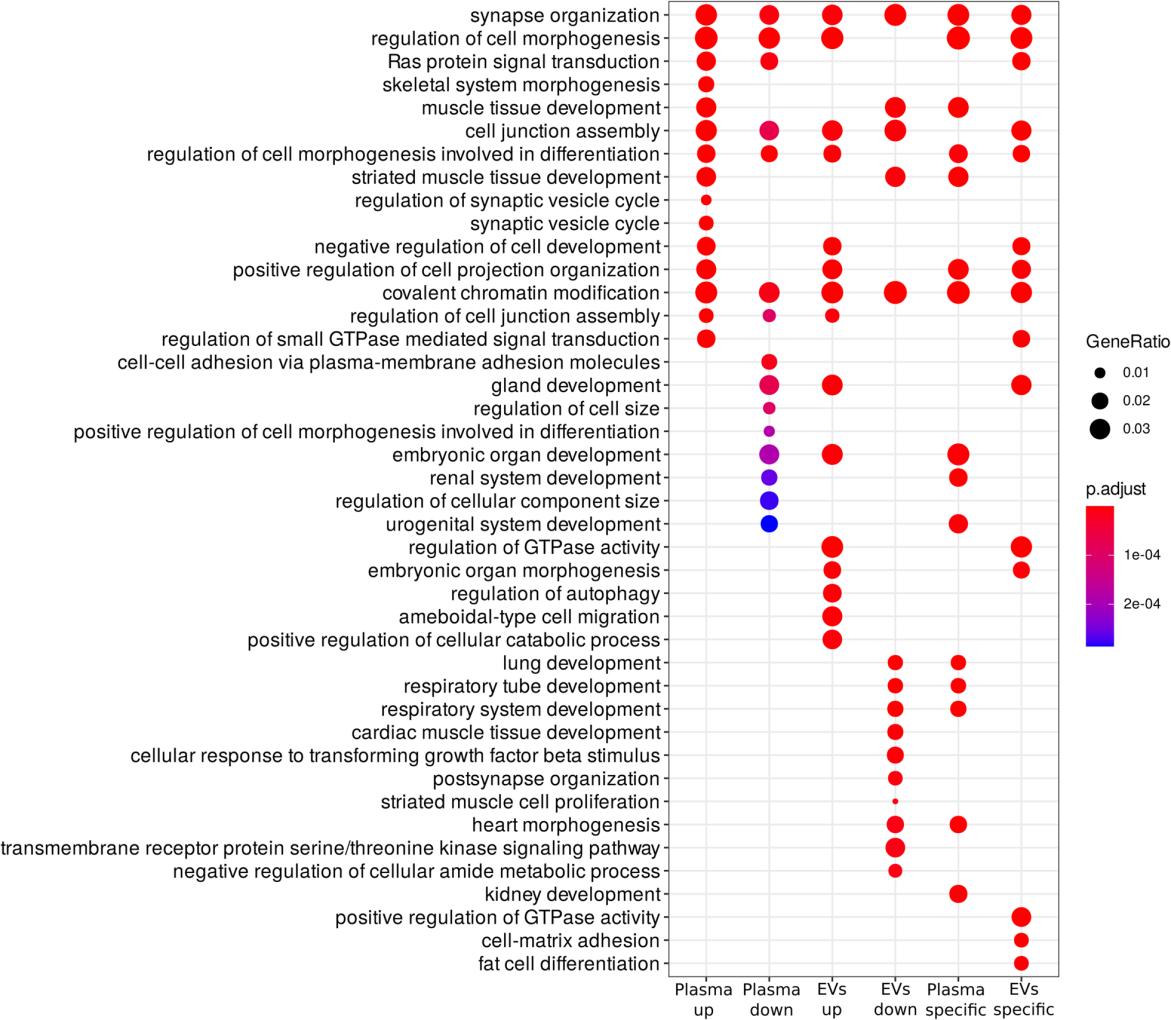
Results of the GO enrichment analysis (top 15 terms for each group) among targets of differentially expressed miRNAs in plasma, extracellular vesicles, and between the two compartments (plasma up—analysis for miRNA up-regulated in plasma, plasma down—analysis for miRNA down-regulated in plasma, EVs up—analysis for miRNA up-regulated in EVs, EVs down—analysis for miRNA down-regulated in EVs, plasma specific—analysis for miRNA which abundance in plasma is higher than in EVs, in comparison to controls, EVs specific—analysis for miRNA which abundance in EVs is higher than in plasma, in comparison to controls)

The top 15 GO terms for each gene set are presented in Fig. 3. Among plasma up- and EVs down-regulated GO terms, ‘muscle tissue development’ and ‘striated muscle tissue development’ are present. In contrast, ‘Cardiac muscle tissue development’ and ‘cellular response to transforming growth factor beta stimulus’, ‘striated muscle cell proliferation’ and ‘heart morphogenesis’ terms are unique for EVs ‘down-regulated” GO. Moreover, when all significant GO were filtered against the “cardiac” term, ontologies such as ‘cardiac muscle cell action potential’, ‘cardiac muscle cell contraction’, ‘cardiac muscle hypertrophy in response to stress’ or ‘cardiac muscle adaptation’ appeared.

**Fig. 4:**
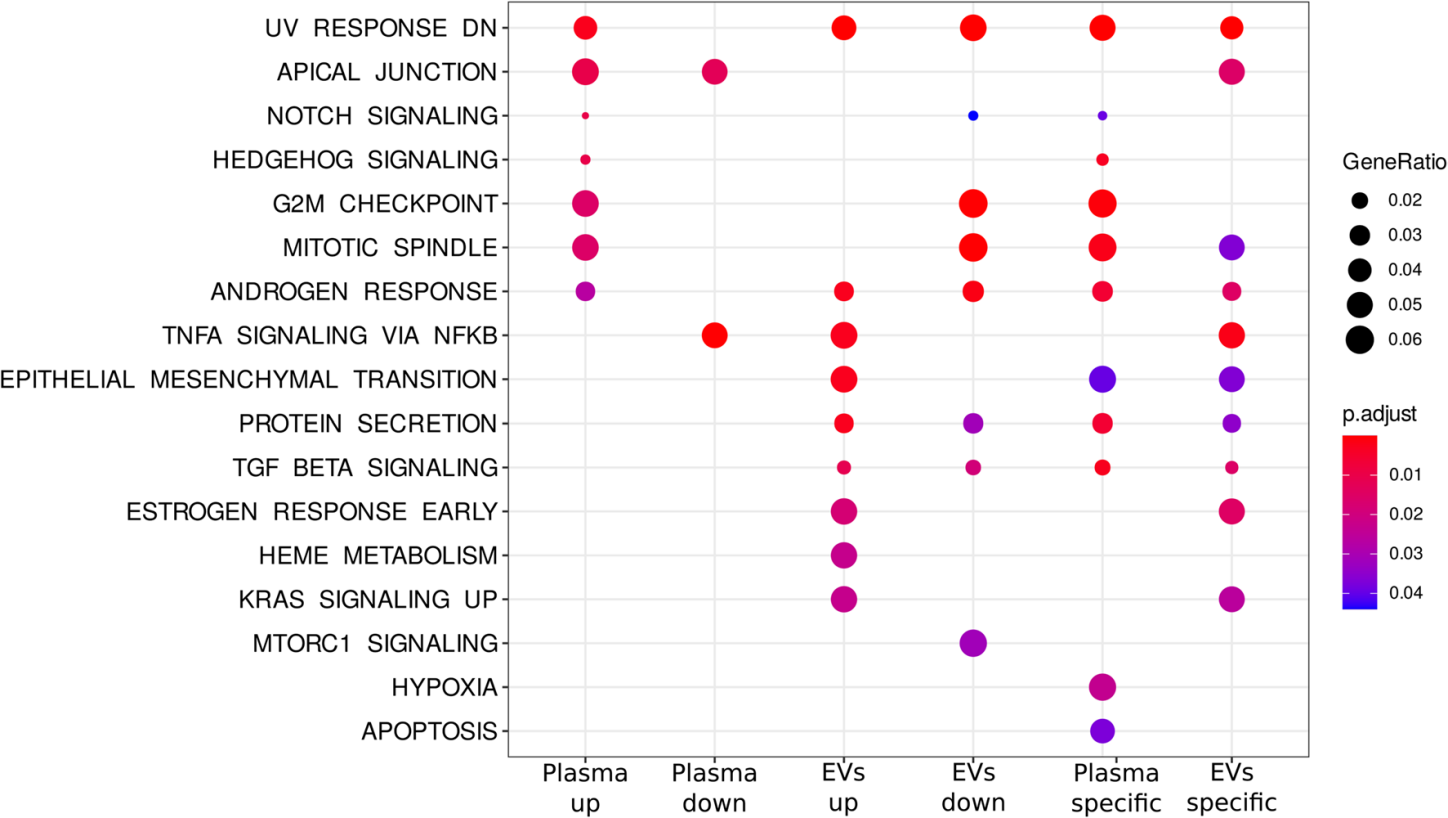
Results of the ‘hallmark’ enrichment analysis among targets of differentially expressed miRNAs in plasma, extracellular vesicles, and between the two compartments (plasma up—analysis for miRNA up-regulated in plasma, plasma down—analysis for miRNA down-regulated in plasma, EVs up—analysis for miRNA up-regulated in EVs, EVs down—analysis for miRNA down-regulated in EVs, plasma specific—analysis for miRNA which abundance in plasma is higher than in EVs, in comparison to controls, EVs specific—analysis for miRNA which abundance in EVs is higher than in plasma, in comparison to controls)

Finally, we looked upon the “hallmark” gene sets from the Molecular Signature Database (MSigDB), to identify the relevant biological processes that are regulated by the target genes of our miRNAs sets. Briefly, our results are presented in Fig. 4. Detailed information about discovered hallmarks are in Additional file 6. ‘Response to UV’ is present in all analyses except plasma down-regulated miRNAs. For plasma up- and EVs down-regulated miRNAs, we found terms related to cell division/DNA damage (‘G2M checkpoint, ‘mitotic spindle’). Moreover, ‘TNFA via NFKB’ is present for plasma down-regulated miRNA and for EVs up-regulated, it is also enriched in EVs of ALL survivors, in comparison to plasma (‘EVs specific’). Beyond the aforementioned, ‘epithelial-mesenchymal transition’ is present for EVs up-regulated, plasma specific and EVs specific. Furthermore, TGFβ signaling is present for EVs up- and down-regulated miRNA, as well as for miRNA with altered distribution between plasma and EVs in comparison to healthy controls. The terms ‘Hypoxia’ and ‘apoptosis’ are unique for these miRNAs, which are enriched in plasma when compared to EVs in ALL survivors (plasma specific). In addition, ‘NOTCH signaling’ is specific for EVs up, plasma down and plasma specific miRNA sets.

Altogether, these results show that both miRNA compartments, vesicular and of total plasma, point to processes that may led to cardiomyopathy development, including TGFβ signaling, EMT and contraction-related issues. However, pathways regulated by differentially expressed miRNAs contained within two compartments are different and thus may diversely contribute to cardiac complications. Additionally, the analysis of differentially distributed miRNAs implies that some miRNA secretory mechanisms might be dysregulated in ALL survivors.

### miRNAs associated with cardiomyopathy

With data on transcriptomic alterations in former ALL survivors, we asked whether our differentially expressed miRNAs are unique for such group or despite different cardiac disease origins, share common features with patients suffering from clinically manifested cardiomyopathies. To achieve that, we used as a validation cohort, the data set of Akat et al. (2014), as this includes samples from people with idiopathic cardiomyopathy (ICM) and dilated cardiomyopathy (DCM). We re-analyzed this data set and compared the differentially expressed miRNAs in plasma and EVs sets from our data with miRNAs differentially expressed between the serum of healthy individuals and patients with ICM or DCM. We found that 14 and 13 differentially expressed miRNAs in the plasma of ALL survivors were also present in the blood plasma of ICM and DCM patients, respectively (Table 3). Similarly, we found 14 and 8 differentially expressed miRNAs in EVs of ALL survivors that were also presented in the blood plasma of ICM and DCM patients, respectively (Table 3). These results suggest that despite echocardiographic measurements not revealing significant pathological functional changes in relatively young ALL survivors, particular miRNAs may be, even at such early point, a good indicator of molecular processes leading later to cardiomyopathy.

**Table 3:** List of differentially expressed miRNAs in plasma or EVs of ALL survivors shared with ICM or DCM datasets

Group	Common DE miRNA
ICM and ALL plasma	miR-208b, miR-3680, miR-202, miR-101, miR-769, miR-511, miR-181b, miR-216a, miR-210, miR-3158, miR-584, miR-455, miR-95, miR-1277
DCM and ALL plasma	miR-1, miR-208b, miR-144, miR-194, miR-511, miR-181b, miR-216a, miR-210, miR-3158, miR-584, miR-455, miR-193a, miR-95
ICM and ALL EVs	miR-199b, miR-148a, miR-200a, miR-361, miR-429, miR-21, miR-132, miR-15b, miR-215, miR-200b, miR-197, miR-10b, miR-29a, miR-143
DCM and ALL EVs	miR-148a, miR-369, miR-1, miR-15b, miR-215, miR-1180, miR-31, miR-29a

### miRNAs and echocardiographic parameters

On applying differential miRNA analysis, we obtained multiple results that suggest the association of circulating miRNAs in plasma and in EVs, with cardiomyopathy. Therefore, we asked whether DE miRNAs are related to cardiac system functioning. To answer that question, we correlated the DE miRNA from each compartment with echocardiographic parameters. We found that many DE miRNAs correlate with indicators of cardiac function (Additional files 7 and 8). Therefore, we decided to test the expression of most variable miRNAs with echocardiographic parameters, in each compartment separately. First, we filtered out all miRNAs with a median expression of 5 pseudocounts in each compartment. Subsequently, using Levene’s test, we selected significant miRNAs (FDR < 0.05) that differ between ALL survivors and controls (Table 4), in plasma and EVs separately. After the aforementioned, we correlated each echocardiographic parameter with those miRNAs.

**Table 4:** Results of the Levene’s test for equality of variances in plasma and EVs between ALL survivors and controls

miRNA	Precursor	p-value	FDR
Plasma			
miR-423-3p	mir-423	1.43E−07	4.85E−05
miR-144-3p	mir-144	1.11E−06	1.88E−04
miR-25-3p	mir-25	5.62E−06	6.33E−04
let-7g-5p	let-7g	2.50E−05	2.01E−03
miR-101-3p	mir-101-2	3.53E−05	2.01E−03
miR-101-3p	mir-101-1	3.56E−05	2.01E−03
miR-342-5p	mir-342	9.09E−05	4.39E−03
miR-501-3p	mir-501	2.32E−04	9.80E−03
miR-532-5p	mir-532	3.80E−04	1.43E−02
miR-16-2-3p	mir-16-2	6.00E−04	1.69E−02
miR-140-3p	mir-140	6.50E−04	1.69E−02
miR-182-5p	mir-182	6.68E−04	1.69E−02
miR-486-5p	mir-486-1	6.95E−04	1.69E−02
miR-486-5p	mir-486-2	7.00E−04	1.69E−02
miR-215-5p	mir-215	1.52E−03	3.42E−02
miR-1180-3p	mir-1180	1.78E−03	3.72E−02
let-7a-5p	let-7a-3	2.09E−03	3.72E−02
let-7a-5p	let-7a-2	2.09E−03	3.72E−02
let-7a-5p	let-7a-1	2.09E−03	3.72E−02
miR-100-5p	mir-100	2.24E−03	3.79E−02
let-7c-5p	let-7c	2.82E−03	4.54E−02
EVs			
miR-144-3p	mir-144	2.73E−06	1.07E−03
miR-7976	mir-7976	5.55E−06	1.09E−03
miR-6747-3p	mir-6747	2.11E−05	2.76E−03
let-7f-5p	let-7f-1	4.38E−05	2.85E−03
let-7a-5p	let-7a-3	5.07E−05	2.85E−03
let-7a-5p	let-7a-1	5.09E−05	2.85E-03
let-7a-5p	let-7a-2	5.09E−05	2.85E−03
let-7f-5p	let-7f-2	6.73E−05	3.30E−03
miR-486-5p	mir-486-1	2.02E−04	7.99E−03
miR-486-5p	mir-486-2	2.04E−04	7.99E−03
let-7c-5p	let-7c	3.63E−04	1.22E−02
miR-501-3p	mir-501	3.75E−04	1.22E−02
miR-26b-5p	mir-26b	4.41E−04	1.33E−02
miR-10b-5p	mir-10b	7.17E−04	2.01E−02
miR-423-3p	mir-423	8.79E−04	2.30E−02
let-7g-5p	let-7g	9.97E−04	2.44E−02
miR-101-3p	mir-101-2	1.29E−03	2.86E−02
miR-197-3p	mir-197	1.32E−03	2.86E−02
miR-101-3p	mir-101-1	1.58E−03	3.26E−02
miR-877-5p	mir-877	1.77E−03	3.47E−02
miR-3613-5p	mir-3613	2.13E−03	3.98E−02

*logFC* log fold change, *logCPM* log counts per million, *FDR* false discovery rate

**Fig. 5:**
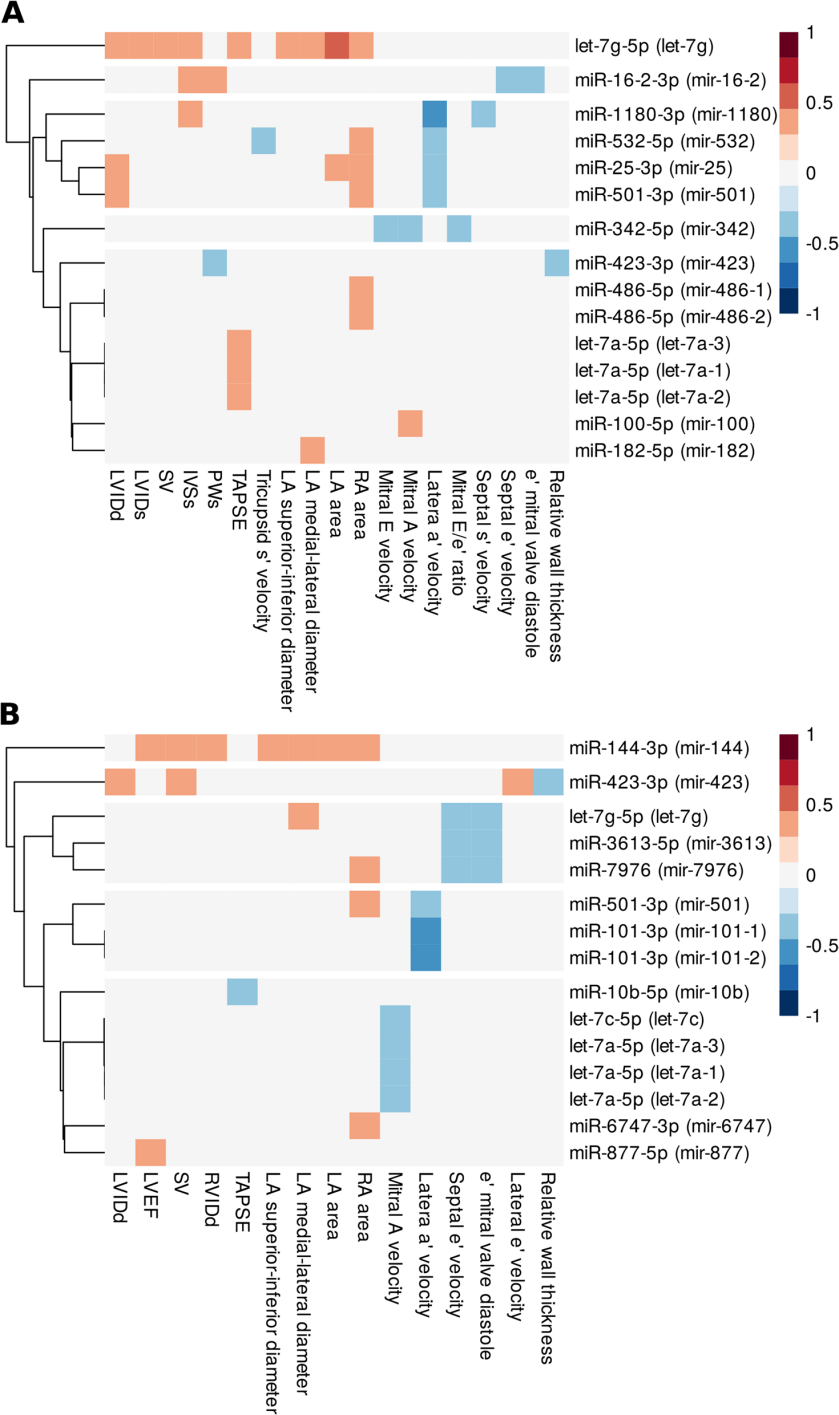
Correlation of the miRNAs, with dissimilar variance in plasma (**A**) and EVs (**B**) between control and ALL survivors, with echocardiography parameters. The miRNAs without significant correlation were removed. Non-significant correlation is presented as white blocks. *LVID* left ventricular internal dimension in systole (s) or diastole (d), *LVEF* left ventricular ejection fraction, *SV* left ventricular stroke volume, *IVSs* septal wall thickness, *PWs* left ventricular posterior wall thickness, *RVID* right ventricular internal dimension, *TAPSE* tricuspid annular plane systolic excursion, *LA* area-left atrial area, *RA* right atrial area

The results of these analyses are presented in Fig. 5. Among plasma miRNA, miR-let-7g-5p was found to be positively correlated with the highest number of echocardiographic parameters, including left ventricle dimensions. Among vesicular miRNA, miR-144-3p was correlated with the highest number of measured variables.

## Discussion

In this paper, we presented diverse lines of evidence that long-term molecular effects of doxorubicin action in ALL survivors include changes of miRNA abundance in circulation that may contribute to the development of cardiomyopathy, a major life-threatening long-term side effect of anthracycline treatment (Mertens et al. 2008; Tukenova et al. 2010; Rosenoff et al. 1975). Long-term cardiomyopathy caused by doxorubicin develops slowly over time without significant functional manifestation. A lot of effort has been made to define the most relevant screening strategies which could help to identify patients at increased risk of developing cardiomyopathy (Lipshultz et al. 2012), but there is still no consensus recommendation considered as the gold standard, Akat et al. (2014) showed that miRNA assessment outperforms the cTnI marker routinely used in heart disease. Therefore, we sequenced miRNA to find those differentially expressed between ALL survivors and healthy subjects. We also characterized miRNA distribution between total plasma and extracellular vesicles. In undertaking this work, we applied several bioinformatic tools to reveal processes ongoing in these subjects, Subsequently, we compared our findings with those previously described in cardiomyopathy patients. Finally, we searched for correlations between identified miRNA and discrete echocardiographic parameters that may be suggestive of incipient cardiac dysfunction.

We confirmed that circulating miRNAs that are differentially expressed in ALL survivors in comparison to healthy people may indicate transcriptional alterations related to cardiac disease development. KEGG enrichment analyses revealed that differentially expressed miRNA in EVs, as well as miRNA that are differentially distributed between plasma and EVs are related to ‘dilated cardiomyopathy’ or ‘arrhythmogenic cardiomyopathy’. Both morphological and functional changes in doxorubicin-induced cardiomyopathy have been reported as similar to those of dilated cardiomyopathy (Lipshultz et al. 1997). This involves the presence of fibrotic areas and myofilaments loss with visible Z-discs disorganization. In advanced pathology, chambers dilation is present with concomitant reduction of ejection fraction and diastolic dysfunction (Benjanuwattra et al. 2020).

We also found other KEGG terms significantly related to DCM, like ‘ERBB signaling’, the pathways of which play key roles in maintaining cardiac structure (Katrien et al. 2007; Rohrbach et al. 2005), as well as in restoring cardiac function after injury (Galindo et al. 2014). Its postnatal disruption leads to dilated cardiomyopathy (Crone et al. 2002) and sensitizes heart to drug-induced toxicity (Popat and Smith 2008). In addition, molecular pathways related to cardiac rhythm and contraction are altered in ALL survivors, which is indicated by KEGG terms ‘arrhythmogenic right ventricular cardiomyopathy’, ‘axon guidance’ or ‘neurotrophin signaling’, which is essential for normal cardiac rhythm through the regulation of cardiac Ca2+ cycling (Feng et al. 2015; Fulgenzi et al. 2015; Miwa et al. 2013). This remains in line with reports showing that doxorubicin affects cardiac electrophysiological properties and may cause various type of arrhythmias (Benjanuwattra et al. 2020). Analysis of gene ontologies further supports this finding, as among significant ontologies, we identified such as related to heart, muscle, cardiocyte, cardiac structures (ventricle, valve), endocardial cushion and cardiac contraction and relaxation.

Our analyses of molecular pathways that are disturbed in ALL survivors revealed that differentially expressed miRNA are involved in the regulation of pathways related to DNA damage, which belongs to canonical effects of doxorubicin action involved in cardiac complications (Qiao et al. 2020) and to pathological cardiac remodeling such as that of NFƘβ and TNFα signaling (Gordon et al. 2011; Wang et al. 2002). Among processes regulated specifically by miRNA differentially expressed in EVs, we identified epithelial-to-mesenchymal- transition (EMT), process linked to therapy-triggered fibrosis (Kalluri and Neilson 2003; Sun et al. 2018) and senescence, which was described as a consequence of genotoxic treatment (Tato-Costa et al. 2016; Baar et al. 2017) and was suggested to reinforce long-term cardiac complications of anticancer treatment (Demaria et al. 2017).

Additionally, both this and GO analysis show that miRNAs encapsulated in EVs are notably involved in TGFβ signaling. TGFβ is a master regulator of EMT (Zavadil et al. 2004; Ferrarelli 2019), the expression of which can be increased in the heart tissue many weeks after doxorubicin treatment (Mancilla et al. 2016). Research has shown that maintaining balance within this pathway is critical for cardiac contractile function, sarcomere kinetics, ion-channel gene expression and cardiomyocyte survival (Umbarkar et al. 2019; Li et al. 2008).

The multiple of differentially expressed miRNAs in our study correlate with cardiac function parameters in ALL survivors, which supports the role of particular miRNAs in cardiac system functioning. However, due to redundancy of miRNA in transcriptomic network and interrelatedness between echocardiographic variables, it is difficult to identify miRNAs predictive of cardiac system function. Therefore, we applied another approach, based on selecting the set of miRNAs, the expression of which is most variable between groups in each of compartments. Strikingly, among the most variable miRNAs in the plasma, let-7g-5p, correlated with the highest number of echocardiographic parameters. This miRNA was reported by Fu et al. as being involved in cardiac cells response to doxorubicin (Fu et al. 2012). Additionally, let-7g was proven to regulate cardiac maturation (Kuppusamy et al. 2015) and to regulate cardiomyocyte survival thorough controlling the expression of PIK3IP1 (Joshi et al. 2016).

We also revealed that similarities in miRNA expression between ALL survivors and patients with advanced, clinically manifested cardiomyopathies exist, despite that subjects in our studied group not having developed any significant cardiac phenotype yet, most probably due to their young age and the short time span between doxorubicin exposure and sample collection.

Of the most variable miRNA set, miR-144, miR-10b and miR-101 are common for plasma and EVs in ALL survivors and ICM/DCM patients. Of these, miR-144-3p, having the highest statistical significance in EVs, is also positively correlated with the highest number of cardiac parameters, including ejection fraction, a parameter that is used to define and to monitor the progress of anthracycline-induced cardiac disease (Leerink et al. 2021). Previous work has confirmed using an animal model that miR-144 is crucial for cardiac function, as its loss worsened the heart failure phenotype, resulting in impaired late remodeling and decreased LVEF (He et al. 2018). Moreover, miR-144 was identified as an important regulatory node in DCM (Huang et al. 2020) and its expression was down-regulated both in samples from DCM patients and in a doxorubicin-induced rodent model of cardiomyopathy (Tao et al. 2019). Another study supporting the important role of miR-144 shows that its loss resulted in ventricular dilation and impaired contractility, whereas intravenous delivery of this miRNA reduced infarcted area and improved cardiac function, including LV fractional shortening, end-systolic volume, end-diastolic volume and ejection fraction (Li et al. 2018). The role of miR-101 in the protection of cardiomyocytes against stress and maintaining cardiac performance upon injury has also been shown (Zhai et al. 2020; Pan et al. 2012), while miR-10b has been proven to regulate the expression of the TBX5 gene, which is necessary for normal heart development (Wang et al. 2014).

Interestingly, only vesicular expression of this miRNA is informative in terms of cardiac functioning. This phenomenon might be related to the specificity of both the EVs packaging and release, as well as EVs uptake—being precise, vesicular miR-144 might have either slightly different cellular origin or target than that circulating outside EVs. Accordingly, previous research has indicated that the abundance of RNA in extracellular space depends, among others, on the cellular system of RNA binding proteins (RBP) that are part of the cell cargo packing and exporting system, shown to be affected by doxorubicin (Statello et al. 2018). In addition, doxorubicin has been found to change the expression of RBPs in rodent cardiomyocytes and in human induced pluripotent stem cell-derived cardiomyocytes (Latorre et al. 2012). Moreover, our analyses reveal that especially miRNA that are differentially distributed between plasma and vesicles (i.e. miRNA that are more or less abundant in EVs than in plasma when compared to healthy people) in ALL survivors indicate processes related to cardiomyopathy. Of note, the term ‘protein secretion’ was present among the enriched hallmarks. This supports the notion that the changes in miRNA presence that we observed are closely linked to the alterations in RNA secretory mechanisms, possibly related to RNA binding proteins.

## Conclusions

In summary, our study has indicated that particular miRNA, including miR-144-3p and let-7g-5p, could be considered as candidates for further studies on cardiac complications in doxorubicin treated cancer survivors. Moreover, we have demonstrated that the compartment that is to be studied with regard to being the source of miRNA should be carefully chosen, as the source of miRNA origin, as well as its destination site may be different in the case of vesicular and total plasma fraction of miRNA. However, both compartments may be useful sources of information on processes that in a long perspective can lead to cardiomyopathy development in former patients treated with anthracyclines, especially if we consider differential distribution between plasma and EVs.

We are aware that it would be of great value if such finding could be confirmed in a follow-up study at later time points in this population, as well as validated in other populations of cancer survivors. The main study limitation is lack of verification of specificity of our findings, i.e. whether similar association between echocardiographic parameters and miRNA expression in blood also exists in healthy individuals or in various forms of cardiomyopathy. This is the first study, to our knowledge, which is aimed at explaining molecular processes leading to distant cardiac effects of doxorubicin treatment in former cancer survivors. It should be underlined that miRNA circulating in blood are accessible and may serve as a source of information on transcriptional processes ongoing in cells. Future studies using samples from patients at different stages of the cardiac diseases caused by anthracyclines would enable validation of the usefulness of the miRNAs that were found to be implicated in cardiac system functioning within this study.

## Supplementary Information

**Additional file 1.** Full list of miRNAs differentially expressed between controls and ALL survivors in blood plasma.

**Additional file 2.** Full list of miRNAs differentially expressed between controls and ALL survivors in EVs.

**Additional file 3.** Full list of miRNAs that are differentially distributed between plasma and EVs in ALL survivors with respect to controls.

**Additional file 4.** Full list of KEGG terms related to miRNA differentially expressed between cases and controls for plasma and EVs compartment and for miRNA that are differentially distributed between compartments.

**Additional file 5.** Full list of GO terms related to miRNA differentially expressed between cases and controls for plasma and EVs compartment and for miRNA that are differentially distributed between compartments.

**Additional file 6.** Full list of hallmarks for differentially expressed miRNAs and for miRNAs that are differentially distributed.

**Additional file 7.** Correlations of differentially expressed miRNAs in plasma with echocardiographic parameters.

**Additional file 8.** Correlations of differentially expressed miRNAs in EVs with echocardiographic parameters.

## Data Availability

The raw RNA sequences, along with raw and normalized counts from miRDeep2 software were deposited in GEO (GSE145176). The plasma miRNA expression data, from ICM and DCM patients, used for the analyses described in this manuscript were obtained from the Gene Expression Omnibus (GEO) database, www.ncbi.nlm.nih.gov/geo (accession no. GSE53081).

## Abbreviations

ALL: Acute lymphoblastic leukemia
ROS: Reactive oxygen species
DSBs: Double-strand breaks
EVs: Extracellular vesicles
HD: Heart disease
DCM: Dilated cardiomyopathy
ICM: Idiopathic cardiomyopathy
HC: Healthy individuals
IHC: Immortalized cardiomyocytes
EMT: Epithelial-to-mesenchymal transition
