# Supplementary material for: MicroRNA composition of plasma extracellular vesicles: a harbinger of late cardiotoxicity of doxorubicin
Source: Mol Med. 2022 Dec 14;28:156. doi: 10.1186/s10020-022-00588-0 (PMC9753431; doi:10.1186/s10020-022-00588-0)
Supplement: Supplementary file 1 — Additional file 1. Full list of miRNAs differentially expressed between controls and ALL survivors in blood plasma. [file 10020_2022_588_MOESM1_ESM.pdf]

miRNA	logFC	logCPM	FDR
hsa-miR-184	-5,358041965	8,897373149	4,94973E-13
hsa-miR-324-5p	-3,275646672	1,897315339	4,59021E-07
hsa-miR-4753-5p	-3,14917422	1,352234825	1,04753E-06
hsa-let-7g-5p	-0,701036923	13,64181028	1,04753E-06
hsa-miR-579-5p	-3,385228555	1,967915881	1,65692E-06
hsa-miR-1-3p	-2,992801913	7,536179085	2,15953E-06
hsa-miR-1-3p	-2,972897538	7,480340293	2,15953E-06
hsa-miR-3140-3p	-3,107606639	1,628845538	4,6746E-06
hsa-miR-3939	-2,335112245	1,270423483	6,21243E-05
hsa-miR-1273c	4,798696823	3,363626748	8,21137E-05
hsa-miR-208b-3p	5,586630038	1,993472182	0,000106949
hsa-miR-6754-3p	-2,463468018	1,33939288	0,000106949
hsa-miR-6858-5p	-3,673852798	1,412978859	0,000142423
hsa-miR-450a-5p	-1,550514497	5,131804829	0,000142423
hsa-miR-450a-5p	-1,550514497	5,131804829	0,000142423
hsa-miR-144-3p	-1,320395847	9,033908983	0,000167305
hsa-miR-451a	0,604510629	17,63728254	0,000178616
hsa-miR-3918	-2,694971678	1,187128495	0,000178616
hsa-miR-6511b-5p	-2,371968414	1,439240533	0,000178616
hsa-miR-6511b-5p	-2,371968414	1,439240533	0,000178616
hsa-miR-423-5p	0,367425227	14,79289896	0,000443261
hsa-miR-188-5p	-2,599597202	1,606140289	0,000443261
hsa-miR-6769b-3p	-2,47133663	1,162687617	0,000443261
hsa-miR-1236-5p	4,76574388	1,647903527	0,000443261
hsa-miR-7976	-1,793093414	4,240397554	0,000443261
hsa-miR-3680-3p	-2,571498198	1,289631944	0,000443261
hsa-miR-3680-3p	-2,571498198	1,289631944	0,000443261
hsa-miR-202-5p	-2,587258999	1,712718357	0,000448333
hsa-miR-3199	3,274108498	2,558486267	0,000541659
hsa-miR-3199	3,274108498	2,558486267	0,000541659
hsa-miR-6885-3p	-2,856512598	1,186415536	0,000649553
hsa-miR-7850-5p	-2,428045708	1,274802492	0,000649553
hsa-miR-6809-5p	4,002657171	2,637575233	0,000953823
hsa-miR-101-3p	-0,722101637	12,28869552	0,000993613
hsa-miR-101-3p	-0,715279726	12,27699951	0,001100105
hsa-miR-6509-5p	2,441635049	2,505588794	0,001100105
hsa-miR-190a-3p	-2,485595633	1,160080722	0,001100105
hsa-miR-3678-3p	-3,12703801	1,173894502	0,001100105
hsa-miR-194-3p	-1,848259388	1,175092908	0,001100105
hsa-miR-6875-5p	-2,413146361	1,430368708	0,001100105
hsa-miR-6859-3p	-1,544023207	1,111887973	0,001100105
hsa-miR-6859-3p	-1,544023208	1,111887973	0,001100105
hsa-miR-6859-3p	-1,544023208	1,111887973	0,001100105
hsa-miR-6859-3p	-1,544023208	1,111887973	0,001100105
hsa-miR-3163	-2,626873782	1,839332381	0,001104688
hsa-miR-6763-5p	4,543139955	2,080751284	0,001221434
hsa-miR-4639-5p	-2,458017175	1,391670301	0,001535395
hsa-miR-769-3p	-4,185658816	1,378921956	0,00154568
hsa-miR-3157-5p	-1,662317426	1,101111943	0,00154568
hsa-miR-4804-5p	-2,6047582	1,736418683	0,00154568
hsa-miR-511-3p	-2,862768683	1,192582931	0,001612453
hsa-miR-1285-5p	-2,029517283	1,241071652	0,001612453
hsa-miR-4474-3p	-2,291432866	1,445257377	0,001615197
hsa-miR-4745-3p	-1,985500586	1,131748297	0,001615197

hsa-miR-6800-5p	2,71257958	1,315101435	0,002148845
hsa-miR-4690-3p	-2,620955216	1,758776996	0,002148845
hsa-miR-6510-3p	-2,430153988	1,149632959	0,002385781
hsa-miR-34a-5p	-2,015291474	1,322258893	0,002573515
hsa-miR-6782-5p	1,825820135	1,085560486	0,002584215
hsa-miR-181b-3p	-2,567034496	1,229556434	0,002877451
hsa-miR-216a-5p	-1,800717521	1,077835344	0,002877451
hsa-miR-942-3p	-2,145317411	1,768354927	0,003128649
hsa-miR-5000-3p	-1,778277215	1,129689327	0,003576135
hsa-miR-3168	2,330207759	8,690054625	0,00362342
hsa-miR-6511a-5p	-2,356936345	1,145807494	0,003907478
hsa-miR-6511a-5p	-2,356936345	1,145807494	0,003907478
hsa-miR-6511a-5p	-2,356936345	1,145807494	0,003907478
hsa-miR-6511a-5p	-2,356936345	1,145807494	0,003907478
hsa-miR-592	2,014839216	1,159784807	0,003907478
hsa-let-7i-5p	-0,372411722	14,62731767	0,004022769
hsa-miR-6819-5p	-2,549713769	1,253706172	0,004049173
hsa-miR-643	-2,36877987	1,408321084	0,004140715
hsa-miR-1295a	-2,112125985	1,137060463	0,004140715
hsa-miR-6772-5p	-3,162125693	1,191319976	0,004373326
hsa-miR-6882-5p	3,506230895	2,558006799	0,004373326
hsa-miR-1973	-11,12681631	6,190337164	0,004373326
hsa-miR-4775	-2,683366045	1,677765544	0,004593605
hsa-miR-210-3p	-1,430918237	3,88437231	0,004985693
hsa-miR-4686	-2,282875542	1,804856617	0,00509626
hsa-miR-6810-3p	-2,737296909	1,147653672	0,005297063
hsa-miR-6883-3p	-1,535563736	1,107737867	0,005351353
hsa-miR-6861-3p	-1,662317688	1,108741655	0,005474701
hsa-miR-4536-5p	4,132720606	1,444882032	0,005542466
hsa-miR-4536-5p	4,132720606	1,444882032	0,005542466
hsa-miR-6761-5p	-1,961411109	1,139900221	0,006651415
hsa-miR-6787-3p	-2,431922392	1,167673258	0,006677702
hsa-miR-9-5p	-1,906935283	3,287361727	0,006677702
hsa-let-7f-2-3p	1,894904944	3,656800384	0,006855893
hsa-miR-9-5p	-1,906935283	3,286524135	0,006976339
hsa-miR-9-5p	-1,906935283	3,286524135	0,006976339
hsa-miR-548h-5p	-1,57990818	1,066169511	0,007304345
hsa-miR-548h-5p	-1,57990818	1,066169511	0,007304345
hsa-miR-548h-5p	-1,57990818	1,066169511	0,007304345
hsa-miR-548h-5p	-1,57990818	1,066169511	0,007304345
hsa-miR-548h-5p	-1,57990818	1,066169511	0,007304345
hsa-miR-29c-5p	2,221155619	2,926167463	0,007407449
hsa-miR-100-5p	-0,867001222	8,820601319	0,00774807
hsa-miR-3158-3p	0,8373293	8,128678499	0,00815234
hsa-miR-3158-3p	0,8373293	8,128678499	0,00815234
hsa-miR-1227-3p	-1,314530127	1,097970106	0,008360735
hsa-miR-1298-5p	-1,514525912	1,152583266	0,008396756
hsa-miR-4635	2,68631507	2,058123166	0,008396756
hsa-miR-4668-5p	-1,717171282	1,381485203	0,008643308
hsa-miR-548at-3p	2,28801285	1,162821891	0,00930713
hsa-miR-5585-3p	-1,781032674	1,083689791	0,00930713
hsa-miR-4784	-1,701759634	1,128177579	0,010711962
hsa-miR-4492	-3,493597147	1,235848899	0,010711962
hsa-miR-3173-3p	2,386713054	1,695635486	0,010952086
hsa-miR-7705	-2,002449831	1,241503914	0,011094739

hsa-miR-4479	-1,469459132	1,081589484	0,011809387
hsa-miR-2277-5p	-3,198257498	1,282804603	0,011809387
hsa-miR-6761-3p	-2,287462498	1,161828144	0,011837361
hsa-miR-4660	-1,144367251	1,137005802	0,012138363
hsa-miR-3183	-1,870162776	1,064589683	0,012190697
hsa-miR-4728-3p	-2,444475969	1,150283082	0,013075725
hsa-miR-584-5p	-0,749879975	9,727931052	0,013425866
hsa-miR-600	1,916184986	1,088814218	0,013442556
hsa-miR-6764-3p	-3,198030245	1,188131316	0,013442556
hsa-miR-3200-5p	-1,793285037	1,636154732	0,013446695
hsa-miR-106a-3p	-1,121069802	1,078575563	0,014001413
hsa-miR-1185-2-3p	-2,45004968	1,191977859	0,014434997
hsa-miR-5701	-2,163287724	1,106035369	0,014523677
hsa-miR-5701	-2,163287724	1,106035369	0,014523677
hsa-miR-5701	-2,163287724	1,106035369	0,014523677
hsa-miR-549a-3p	-2,967108015	1,227452877	0,015654638
hsa-miR-4672	-1,142654266	1,414400342	0,015654638
hsa-miR-3131	2,652617129	1,39398228	0,015654638
hsa-miR-365a-5p	3,020581723	1,81766866	0,016175662
hsa-miR-3176	-1,991639005	1,830773483	0,017561513
hsa-miR-181b-2-3p	-1,975505332	1,160968138	0,018088
hsa-miR-627-3p	-1,735309347	1,37426863	0,022458195
hsa-miR-7975	6,482246401	2,237723449	0,023425897
hsa-miR-190a-5p	-1,143511154	1,131556233	0,02360696
hsa-miR-4741	-1,827724596	1,355926644	0,023993801
hsa-let-7f-5p	-0,476861284	13,47043622	0,024484976
hsa-miR-877-3p	2,176464534	1,133667354	0,02480728
hsa-miR-126-3p	-0,477484025	14,26679386	0,025011144
hsa-miR-2355-3p	-1,541488395	3,283798765	0,025651488
hsa-miR-500a-5p	-1,386494	1,086466587	0,026074878
hsa-miR-3614-5p	1,930454844	2,536658461	0,026840069
hsa-miR-449a	-1,978315448	1,076923761	0,026840069
hsa-miR-6743-3p	-1,340912233	1,048143166	0,026840069
hsa-miR-3677-3p	-1,323629882	1,121120125	0,028640365
hsa-miR-3150b-5p	-2,33548723	1,105337043	0,028663906
hsa-miR-12114	2,388644188	1,156255975	0,028663906
hsa-miR-3942-5p	-1,692162953	1,161774426	0,028685217
hsa-miR-3175	-1,585140283	1,204839177	0,029176374
hsa-miR-1229-3p	-1,495058226	2,836860909	0,03037368
hsa-miR-4726-5p	-3,050323464	1,23214329	0,030434202
hsa-miR-6750-5p	1,739637591	2,335062955	0,030750877
hsa-miR-455-3p	-1,832939766	1,136677778	0,030784751
hsa-miR-6777-5p	-1,043333117	1,162392232	0,031090007
hsa-miR-193a-3p	-1,80903201	1,193135088	0,032713237
hsa-miR-3127-5p	1,703570435	2,079386525	0,03373859
hsa-miR-548b-5p	-1,572535399	1,28191494	0,033875836
hsa-miR-135a-2-3p	5,077916567	2,01945885	0,034004215
hsa-miR-500b-5p	-1,331920923	1,084058435	0,034221581
hsa-miR-4437	-1,451898284	1,137197138	0,034700425
hsa-miR-141-5p	-1,881504191	1,126884486	0,034700425
hsa-miR-3936	2,481542504	1,504084839	0,035472181
hsa-miR-4448	-3,490733915	2,758998915	0,035472181
hsa-miR-1273h-5p	-1,578390465	3,216804746	0,035472181
hsa-miR-95-3p	1,181722996	3,840642052	0,035472181
hsa-miR-3117-3p	2,086682891	1,148386032	0,036145676

hsa-miR-211-5p	-1,13578246	1,096846361	0,036422089
hsa-miR-423-3p	-0,602785678	11,31732469	0,036516297
hsa-miR-642a-5p	2,295473433	1,120097221	0,037156164
hsa-miR-4797-5p	-1,058855699	1,111637612	0,037488364
hsa-miR-4689	-1,638814541	1,057848766	0,03757038
hsa-miR-548t-5p	-2,103141801	1,375612899	0,039690075
hsa-miR-7108-5p	-1,591883733	1,108128301	0,039690075
hsa-miR-4525	1,794317105	1,236078572	0,039957644
hsa-miR-6845-5p	2,02224026	1,141944603	0,040587845
hsa-miR-1277-3p	-1,754015385	1,499584982	0,042722364
hsa-miR-4659b-5p	1,128717611	1,081390643	0,042793285
hsa-miR-548ae-3p	-1,096505171	1,042726068	0,043629891
hsa-miR-548ae-3p	-1,096505171	1,042726068	0,043629891
hsa-miR-4503	-0,893521375	1,083377774	0,044242535
hsa-miR-6780a-3p	1,605311718	1,245844737	0,044348534
hsa-miR-6796-3p	3,132716185	1,201105224	0,044348534
hsa-miR-6770-5p	-1,328911476	1,050060474	0,044348534
hsa-miR-6770-5p	-1,328911476	1,050060474	0,044348534
hsa-miR-6770-5p	-1,328911476	1,050060474	0,044348534
hsa-miR-4446-5p	-3,984378188	1,359702743	0,045323698
hsa-miR-450a-2-3p	-1,621283995	1,596929292	0,045323698
hsa-miR-653-5p	2,133256193	1,350703623	0,045323698
hsa-miR-4653-3p	2,220710118	1,141317975	0,045323698
hsa-miR-146a-5p	-0,668447081	11,00101586	0,045323698
hsa-miR-6726-5p	1,903868252	1,096480954	0,045576545
hsa-miR-6130	-0,927290613	4,10063547	0,046124728
hsa-miR-6129	2,405730887	1,242113988	0,046124728
hsa-miR-9-3p	-1,685827397	2,698481212	0,046124728
hsa-miR-9-3p	-1,685827397	2,698434908	0,046124728
hsa-miR-9-3p	-1,685827397	2,698434908	0,046124728
hsa-miR-4433a-5p	-1,795207138	1,168315306	0,046124728
hsa-miR-548ay-3p	-1,583230216	1,489215966	0,047130432
hsa-miR-4779	-0,897589633	1,123439674	0,04715978
hsa-miR-339-5p	-1,244066977	3,280696956	0,047755384
hsa-miR-5588-5p	-1,762222925	1,42211501	0,048372069
hsa-miR-4670-5p	-1,106205637	1,050757069	0,049562193
hsa-miR-616-3p	-1,633943619	1,639496471	0,049562193
