# Supplementary material for: MicroRNA composition of plasma extracellular vesicles: a harbinger of late cardiotoxicity of doxorubicin
Source: Mol Med. 2022 Dec 14;28:156. doi: 10.1186/s10020-022-00588-0 (PMC9753431; doi:10.1186/s10020-022-00588-0)
Supplement: Supplementary file 2 — Additional file 2. Full list of miRNAs differentially expressed between controls and ALL survivors in EVs. [file 10020_2022_588_MOESM2_ESM.pdf]

miRNA	logFC	logCPM	FDR
hsa-miR-221-5p	3,766198205	4,657141088	6,47643E-08
hsa-miR-199a-3p	1,721927901	9,299212138	4,62629E-07
hsa-miR-199b-3p	1,722104718	9,298152539	4,62629E-07
hsa-miR-199a-3p	1,722104718	9,298152539	4,62629E-07
hsa-miR-203a-3p	3,923788693	5,776152847	5,76621E-07
hsa-miR-574-5p	3,100986116	3,979276435	4,88866E-06
hsa-miR-148a-5p	-2,639351025	5,911248408	5,68184E-06
hsa-miR-200a-3p	2,287533759	8,17799454	6,88948E-06
hsa-miR-145-5p	6,219467527	4,239134382	7,21305E-06
hsa-miR-378i	1,890936627	6,074164158	2,12846E-05
hsa-miR-199b-5p	2,711199703	4,224475002	5,80569E-05
hsa-miR-374b-5p	2,129418617	5,806161387	5,80569E-05
hsa-miR-548au-5p	-5,566822508	3,818951199	9,43065E-05
hsa-miR-548am-5p	-5,527560438	3,823900574	0,000100849
hsa-miR-548o-5p	-5,460617954	3,823650109	0,000110194
hsa-miR-548c-5p	-5,460617954	3,823650109	0,000110194
hsa-miR-200c-3p	2,22080145	6,841394971	0,000135505
hsa-miR-6726-3p	3,689821851	2,610595676	0,000234237
hsa-miR-361-3p	1,565572057	6,572709585	0,000888174
hsa-miR-4446-3p	1,882115777	7,411483694	0,000908064
hsa-miR-30b-5p	1,957118804	4,6425128	0,000950942
hsa-miR-429	2,604405374	3,77687453	0,001123345
hsa-miR-21-3p	2,407793244	3,830972013	0,001182196
hsa-miR-369-5p	2,163284132	4,402670878	0,00161899
hsa-miR-1-3p	2,079524252	7,536179085	0,002056309
hsa-miR-132-5p	1,780938193	4,340823398	0,002162809
hsa-miR-374c-5p	-3,061856305	2,292456885	0,002313722
hsa-miR-1301-3p	-1,446246367	6,10379695	0,003327299
hsa-miR-15b-3p	-1,521693118	6,742083583	0,003472884
hsa-miR-215-5p	1,151229139	9,0039711	0,003826616
hsa-miR-23b-3p	1,685593582	4,816828539	0,003968797
hsa-miR-1-3p	1,962302544	7,480340293	0,003968797
hsa-miR-629-3p	-4,761709626	1,801945954	0,005391448
hsa-miR-141-3p	2,192343379	3,978540584	0,006145451
hsa-miR-9983-3p	6,010338118	2,473055883	0,007648433
hsa-miR-200b-3p	1,965047061	7,674243797	0,007801684
hsa-miR-30c-5p	1,076316669	7,433314996	0,01183921
hsa-miR-30c-5p	1,076213138	7,43273547	0,01183921
hsa-miR-197-3p	-1,413586837	4,247700238	0,017438603
hsa-miR-1180-3p	0,832058505	8,091917457	0,021593823
hsa-miR-660-5p	1,628480759	3,568168141	0,021593823
hsa-miR-500a-5p	-1,711097545	1,086466587	0,025605647
hsa-miR-1910-5p	-2,314244172	1,158762022	0,029105742
hsa-miR-500b-5p	-1,704726735	1,084058435	0,0330032
hsa-miR-10b-5p	-0,739469036	10,5952229	0,038672826
hsa-miR-31-5p	5,333374607	3,15596594	0,040586069
hsa-miR-29a-3p	0,816174396	8,690231549	0,041017483
hsa-miR-23a-3p	1,203262069	6,364700123	0,045865336
hsa-miR-143-5p	2,676989335	2,495913078	0,049873163
