# Supplementary material for: MicroRNA composition of plasma extracellular vesicles: a harbinger of late cardiotoxicity of doxorubicin
Source: Mol Med. 2022 Dec 14;28:156. doi: 10.1186/s10020-022-00588-0 (PMC9753431; doi:10.1186/s10020-022-00588-0)
Supplement: Supplementary file 3 — Additional file 3. Full list of miRNAs that are differentially distributed between plasma and EVs in ALL survivors with respect to controls. [file 10020_2022_588_MOESM3_ESM.pdf]

Table3A3

miRNA	logFC	logCPM	FDR
hsa-miR-1973	-11,63305173	6,190337164	0,004156943
hsa-miR-9983-3p	-6,882310991	2,473055883	0,002430438
hsa-miR-145-5p	-6,842180372	4,239134382	0,000137576
hsa-miR-184	-6,218459917	8,897373149	1,51023E-10
hsa-miR-1-3p	-5,072326166	7,536179085	1,17923E-09
hsa-miR-1-3p	-4,935200082	7,480340293	2,32849E-09
hsa-miR-769-3p	-4,899550833	1,378921956	0,004156943
hsa-miR-6774-3p	-4,079970523	2,261537435	0,013881166
hsa-miR-6726-3p	-4,067367746	2,610595676	0,009463637
hsa-miR-3680-3p	-4,024218969	1,289631944	0,000137576
hsa-miR-3680-3p	-4,024218969	1,289631944	0,000137576
hsa-miR-181b-3p	-3,940999122	1,229556434	0,002529875
hsa-miR-579-5p	-3,69246251	1,967915881	0,000137576
hsa-miR-221-5p	-3,639752296	4,657141088	0,000137576
hsa-miR-6810-3p	-3,633769994	1,147653672	0,004156943
hsa-miR-203a-3p	-3,486585024	5,776152847	0,002860986
hsa-miR-199b-5p	-3,434274118	4,224475002	0,000290789
hsa-miR-6772-5p	-3,257799673	1,191319976	0,042053096
hsa-miR-6875-5p	-3,187923324	1,430368708	0,002944083
hsa-miR-429	-3,052472982	3,77687453	0,009028742
hsa-miR-4804-5p	-2,923877534	1,736418683	0,017092883
hsa-miR-6885-3p	-2,918342869	1,186415536	0,011602325
hsa-miR-643	-2,907483943	1,408321084	0,020915789
hsa-miR-4741	-2,880646361	1,355926644	0,016311499
hsa-miR-3140-3p	-2,854506114	1,628845538	0,002379704
hsa-miR-574-5p	-2,782794676	3,979276435	0,006973093
hsa-miR-369-5p	-2,761612394	4,402670878	0,004400568
hsa-miR-942-3p	-2,73323065	1,768354927	0,011602325
hsa-miR-202-5p	-2,713405803	1,712718357	0,011602325
hsa-miR-4753-5p	-2,702024617	1,352234825	0,000509459
hsa-miR-660-5p	-2,674139512	3,568168141	0,003162205
hsa-miR-4474-3p	-2,605150816	1,445257377	0,01498526
hsa-miR-3939	-2,559095695	1,270423483	0,000445158
hsa-miR-190a-3p	-2,554385717	1,160080722	0,015697433
hsa-let-7c-3p	-2,551520892	1,290418628	0,033801844
hsa-miR-6769b-3p	-2,425108401	1,162687617	0,011602325
hsa-miR-3918	-2,407486039	1,187128495	0,012566488
hsa-miR-6858-5p	-2,390013215	1,412978859	0,027877339
hsa-miR-361-3p	-2,362642838	6,572709585	0,000198885
hsa-miR-374b-5p	-2,321228418	5,806161387	0,003789281
hsa-miR-6510-3p	-2,272467754	1,149632959	0,040753288
hsa-miR-548b-5p	-2,267705732	1,28191494	0,040550843
hsa-miR-6777-5p	-2,262205671	1,162392232	0,007668127
hsa-miR-200c-3p	-2,25514797	6,841394971	0,012566488
hsa-miR-324-5p	-2,230339105	1,897315339	0,019597132
hsa-miR-188-5p	-2,167425386	1,606140289	0,033281142
hsa-miR-7850-5p	-2,085356856	1,274802492	0,016993491
hsa-miR-6511b-5p	-2,055797803	1,439240533	0,018668523
hsa-miR-6511b-5p	-2,055797803	1,439240533	0,018668523
hsa-miR-30b-5p	-2,026470736	4,6425128	0,026915932
hsa-miR-23b-3p	-1,991267852	4,816828539	0,021669009
hsa-miR-199b-3p	-1,982099512	9,298152539	9,01781E-05
hsa-miR-199a-3p	-1,982099512	9,298152539	9,01781E-05
hsa-miR-199a-3p	-1,976903186	9,299212138	9,01781E-05

hsa-miR-6754-3p	-1,899971003	1,33939288	0,007668127
hsa-miR-4446-3p	-1,86536997	7,411483694	0,03594229
hsa-miR-4479	-1,855258605	1,081589484	0,035143937
hsa-miR-23a-3p	-1,746107904	6,364700123	0,02282789
hsa-miR-1227-3p	-1,655327508	1,097970106	0,02169912
hsa-miR-190a-5p	-1,54672344	1,131556233	0,039624784
hsa-miR-4672	-1,418551205	1,414400342	0,027926616
hsa-miR-194-3p	-1,281963579	1,175092908	0,04257828
hsa-miR-584-5p	-1,223368018	9,727931052	0,005464819
hsa-miR-6859-3p	-1,178263799	1,111887973	0,040550843
hsa-miR-6859-3p	-1,178263798	1,111887973	0,040550843
hsa-miR-6859-3p	-1,178263798	1,111887973	0,040550843
hsa-miR-6859-3p	-1,178263798	1,111887973	0,040550843
hsa-miR-100-5p	-1,12338675	8,820601319	0,02819891
hsa-miR-146a-5p	-1,032472316	11,00101586	0,040550843
hsa-let-7g-5p	-0,727844672	13,64181028	0,00089739
hsa-miR-21-5p	-0,712634421	14,19420119	0,032656486
hsa-miR-25-3p	-0,537006565	14,08476723	0,027365818
hsa-let-7i-5p	-0,45724866	14,62731767	0,026915932
hsa-miR-15b-3p	1,666841238	6,742083583	0,035143937
hsa-miR-483-3p	2,090447721	3,714413727	0,040753288
hsa-miR-148a-5p	2,292580509	5,911248408	0,011602325
hsa-miR-6782-5p	2,40727863	1,085560486	0,00700289
hsa-miR-6800-5p	2,506950683	1,315101435	0,048834848
hsa-miR-6509-5p	2,521684789	2,505588794	0,030578972
hsa-miR-592	2,605011471	1,159784807	0,011602325
hsa-miR-374c-5p	3,49890085	2,292456885	0,013537308
hsa-miR-3173-3p	3,67405218	1,695635486	0,007349496
hsa-miR-365a-5p	3,927391974	1,81766866	0,039295199
hsa-miR-3199	3,984179514	2,558486267	0,003789281
hsa-miR-3199	3,984179514	2,558486267	0,003789281
hsa-miR-1236-5p	4,224269731	1,647903527	0,035143937
hsa-miR-6796-3p	4,566398923	1,201105224	0,02169912
hsa-miR-1273c	4,892039365	3,363626748	0,004043198
hsa-miR-629-3p	5,564996539	1,801945954	0,011220585
hsa-miR-6763-5p	6,433830949	2,080751284	0,000739027
hsa-miR-548au-5p	7,033981222	3,818951199	9,42012E-05
hsa-miR-548c-5p	7,625177747	3,823650109	1,75367E-05
hsa-miR-548o-5p	7,625177747	3,823650109	1,75367E-05
hsa-miR-548am-5p	7,755738633	3,823900574	1,75367E-05
hsa-miR-208b-3p	7,868964107	1,993472182	1,75367E-05
