# Supplementary figures and images for: MicroRNA composition of plasma extracellular vesicles: a harbinger of late cardiotoxicity of doxorubicin
Source: Mol Med. 2022 Dec 14;28:156. doi: 10.1186/s10020-022-00588-0 (PMC9753431; doi:10.1186/s10020-022-00588-0)

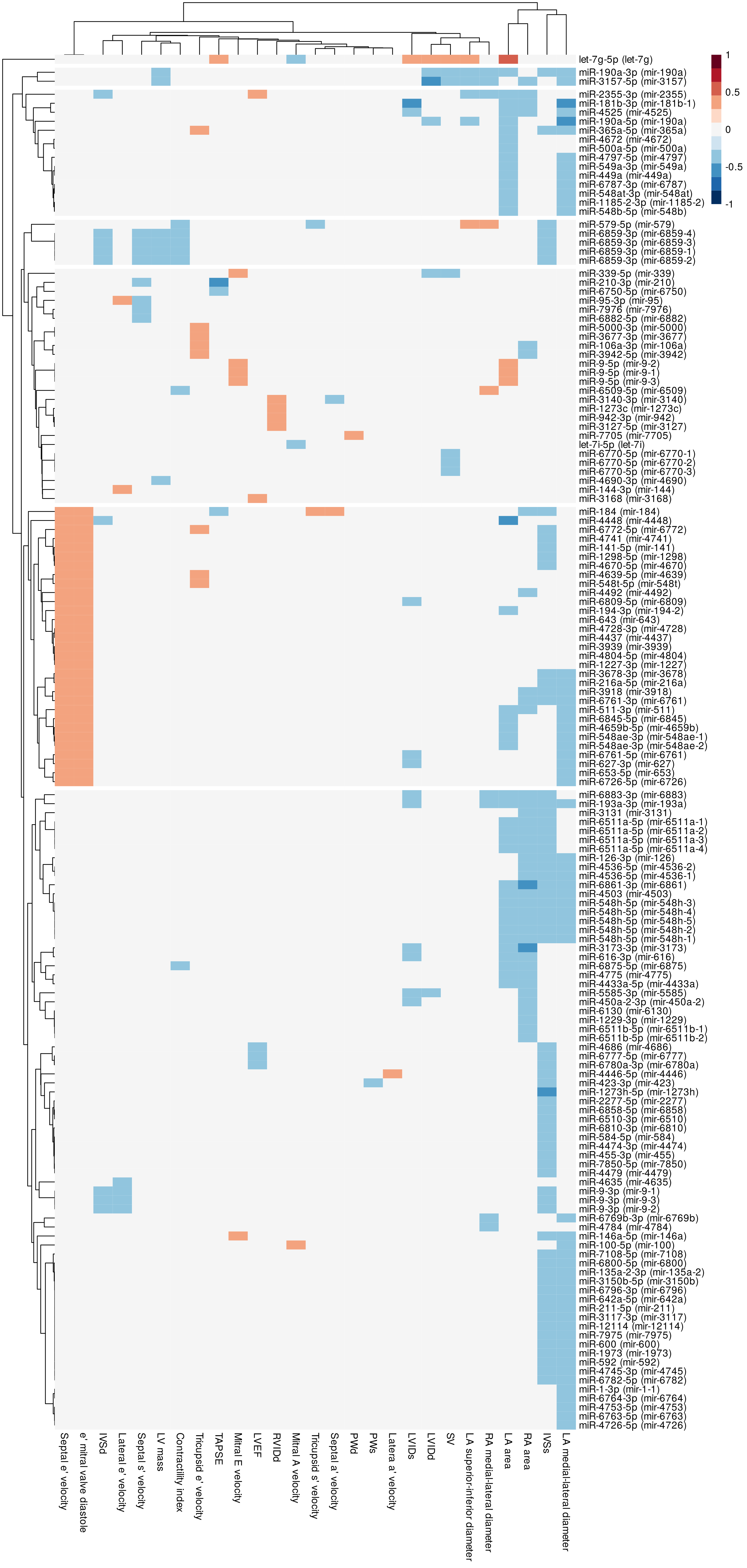

Supplement: Supplementary file 7 — Additional file 7. Correlations of differentially expressed miRNAs in plasma with echocardiographic parameters. [file 10020_2022_588_MOESM7_ESM.png]

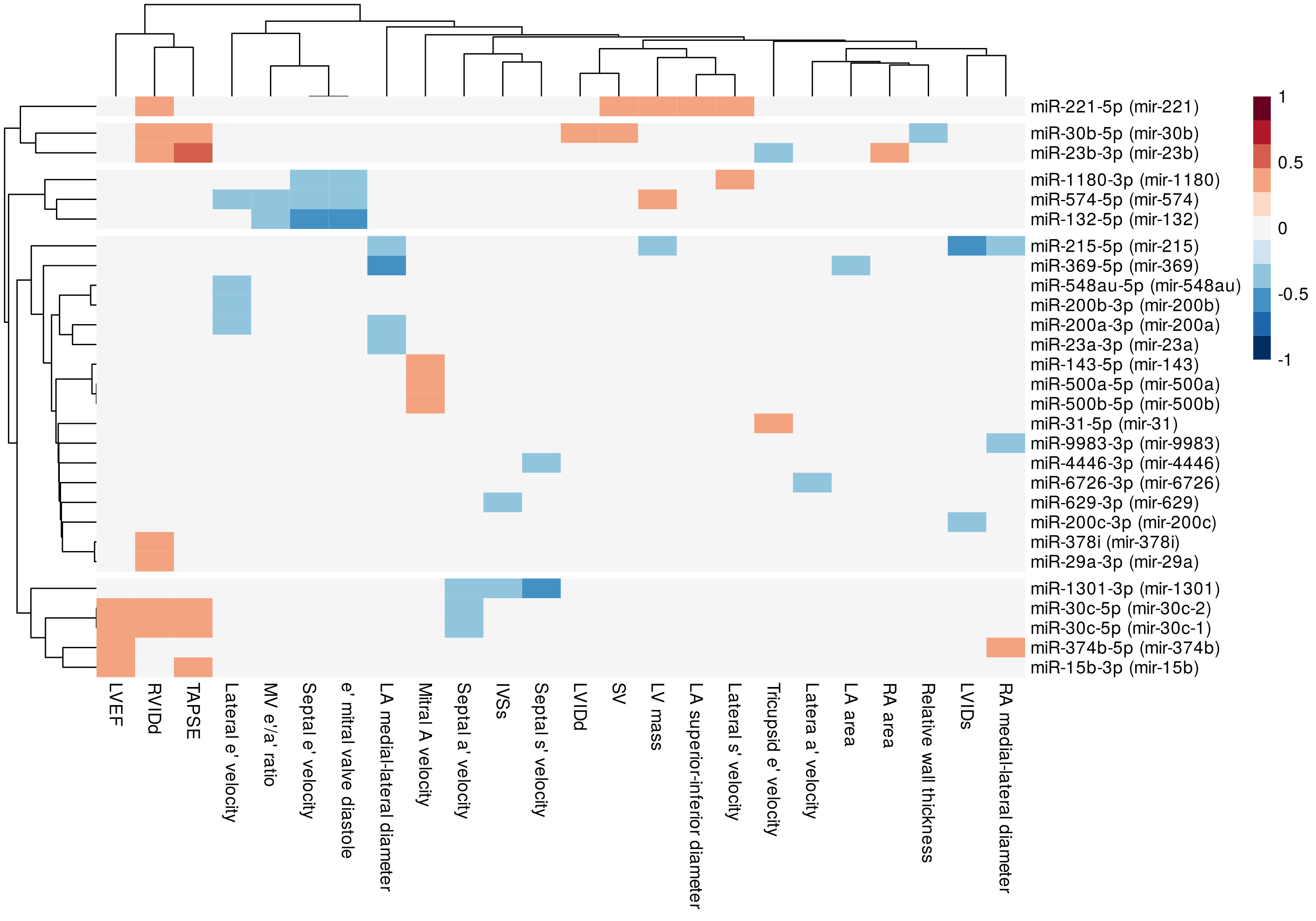

Supplement: Supplementary file 8 — Additional file 8. Correlations of differentially expressed miRNAs in EVs with echocardiographic parameters. [file 10020_2022_588_MOESM8_ESM.png]
